# Genetic Characterization of Avian Influenza A (H11N9) Virus Isolated from Mandarin Ducks in South Korea in 2018

**DOI:** 10.3390/v12020203

**Published:** 2020-02-12

**Authors:** Hien Thi Tuong, Ngoc Minh Nguyen, Haan Woo Sung, Hyun Park, Seon-Ju Yeo

**Affiliations:** 1 Zoonosis Research Center, Department of Infection Biology, School of Medicine, Wonkwang University, Iksan 570-749, Korea; tuonghien23@gmail.com (H.T.T.); minhngoc.adachi@gmail.com (N.M.N.); 2 College of Veterinary Medicine, Kangwon National University, Chuncheon 200-701, Korea; sunghw@kangwon.ac.kr

**Keywords:** novel avian influenza virus isolate, H11N9, South Korea, Japan, Mandarin duck

## Abstract

In July 2018, a novel avian influenza virus (A/Mandarin duck/South Korea/KNU18-12/2018(H11N9)) was isolated from Mandarin ducks in South Korea. Phylogenetic and molecular analyses were conducted to characterize the genetic origins of the H11N9 strain. Phylogenetic analysis indicated that eight gene segments of strain H11N9 belonged to the Eurasian lineages. Analysis of nucleotide sequence similarity of both the hemagglutinin (HA) and neuraminidase (NA) genes revealed the highest homology with A/duck/Kagoshima/KU57/2014 (H11N9), showing 97.70% and 98.00% nucleotide identities, respectively. Additionally, internal genes showed homology higher than 98% compared to those of other isolates derived from duck and wild birds. Both the polymerase acidic (PA) and polymerase basic 1 (PB1) genes were close to the H5N3 strain isolated in China; whereas, other internal genes were closely related to that of avian influenza virus in Japan. A single basic amino acid at the HA cleavage site (PAIASR↓GLF), the lack of a five-amino acid deletion (residue 69–73) in the stalk region of the NA gene, and E627 in the polymerase basic 2 (PB2) gene indicated that the A/Mandarin duck/South Korea/KNU18-12/2018(H11N9) isolate was a typical low-pathogenicity avian influenza. In vitro viral replication of H11N9 showed a lower titer than H1N1 and higher than H9N2. In mice, H11N9 showed lower adaptation than H1N1. The novel A/Mandarin duck/South Korea/KNU18-12/2018(H11N9) isolate may have resulted from an unknown reassortment through the import of multiple wild birds in Japan and Korea in approximately 2016–2017, evolving to produce a different H11N9 compared to the previous H11N9 in Korea (2016). Further reassortment events of this virus occurred in PB1 and PA in China-derived strains. These results indicate that Japanese- and Chinese-derived avian influenza contributes to the genetic diversity of A/Mandarin duck/South Korea/KNU18-12/2018(H11N9) in Korea.

## 1. Introduction

Influenza viruses are highly genetically diverse because of the accumulation of mutations, frequent reassortment among gene segments, host switching ability, and consistent antigenic drift or shift [1,2]. Based on the presence of the 18 hemagglutinin (HA) and 11 neuraminidase (NA) surface glycoproteins and their combination, influenza A viruses have been categorized into subtypes [3]. Particularly, H1–H3, H5–H7, H9, and N1–N2; N6–N9 strains have been detected in multiple hosts such as human, poultry, and swine, whereas H8, and H11–H16; N3–N5 only cause disease in poultry. H17–H18; N10–N11 is only found in bats [4]. 

Unlike various combinations such as H2N2, H3N2, and H1N1, which threaten human health, H11N9 strains have mostly unknown effects for either avians or humans. H11N9 strains were first isolated in Europe (Great Britain) in 1982 [5], after which they were continuously detected through worldwide surveillance, showing low virulence in avians such as in Australia in 1987 and then in 2006, in Europe in 2002–2014 [6,7,8,9,10]; southern Africa in 2008–2009 [11]; Asian countries in 2009 to 2011–2012 [12,13,14]; USA in 2007 [15]; and for the first time in South America in 2014 [16] followed by in 2015 [17]. 

Based on the Global Initiative on Sharing All Influenza Data (GISAID) data, few cases have been detected in South Korea as the influenza H11 subtype, which were H11N4 [18], H11N6, and H11N9. H11N9 with its complete full eight gene segment was reported by Le et al in 2017, from 2006 to date [19].

Some studies have described that H11N9 isolated in China can replicate not only in avian but also in mammalian cells [20], and even in mice lungs without prior adaption [21,22]. Furthermore, the H7N9 strain, which is one of the few avian influenza viruses (AIVs) that have crossed the species barrier and caused significant human morbidity and mortality, was reported to possess NA originating from the H11N9 or H2N9 strains [23,24]. Additionally, an H11 antibody was found on human hunters, suggesting a potent risk of direct transmission of AIV to humans [25].

Therefore, routine surveillance of H11N9 is required, although no human H7N9 strains have been found in Korea. Notably, the H11N6/KU/2/2001 strain exclusively infects swine in Korea. As influenza virus has regularly evolved to infect various hosts, we examined the genetic diversity of AIV gene pools. In this study, the full genome of an H11N9 strain isolated from the feces of Mandarin ducks during surveillance in July 2018 in Korea was analyzed and characterized. 

## 2. Materials and Methods

### 2.1. Sample Collection

Fresh feces were collected from wild birds using sterile swabs in South Korea in July 2018. The collected samples were stored at 2–8 °C, and then shipped to the laboratory within 12 h for further analysis.

### 2.2. Virus Isolation from the Samples

The fecal samples were resuspended in phosphate-buffered saline (pH 7.4) supplemented with antibiotics (100 U/μL of penicillin and 100 mg/μL of streptomycin) and thoroughly mixed by vortexing, followed by centrifugation at 3000 rpm for 10 min at 4 °C. The supernatants were filtered through hydrophilic polyether sulfone membrane filters (0.45 µm pore size; GVS Syringe, Novatech, Kingwood, TX, USA). The filtered aqueous solution was inoculated into the allantoic cavities of specific pathogen-free (SPF) 10-day-old embryonated chicken eggs (Seng-Jin Inc., Eumsung, Korea), which were then incubated at 37 °C for 72 h under humidified conditions and chilled at 4 °C overnight. The allantoic fluids from the inoculated eggs were checked for the presence of influenza A virus by an HA assay as previously described. 

### 2.3. RNA Extraction and Subtyping

Viral RNA was extracted directly from allantoic fluid samples positive for HA activity using the RNeasy Mini Kit (Macherey-Nagel, Düren, Germany) according to the manufacturer’s instructions. The RNA was eluted in 60 μL of RNase-free water to which 20 units of RNase inhibitor was added for storage at −80 °C until use. As a negative control, sterile water was added rather than the specimen.

Additionally, to evaluate influenza growth and determine the subtype, reverse transcription polymerase chain reaction (RT-PCR) for influenza A virus was performed by amplification of the matrix gene-coding sequence following World Health Organization guidelines [26]. RT-PCR was performed using a Probe RT-PCR Kit (QIAGEN, Hilden, Germany) and the cycle threshold (Ct) values were determined using a CFX96 Real-Time PCR Detection System (Bio-Rad, Hercules, CA, USA). Subtyping was determined by well-known primers and probes as previously described [27]. Bird identification was performed using primers for the mitochondrial cytochrome oxidase gene with DNA isolated from the fecal samples as previously described [28].

### 2.4. Sequencing Using Illumina HiSeq X Method for Next-Generation Sequencing (NGS)

NGS was conducted by GnCBIO (Daejeon, Korea) following the Hiseq X method as previously reported [29]. Briefly, influenza RNA was detected using an RNA 6000 pico kit (Agilent Technologies, Santa Clara, CA, USA), and the RNA concentration was measured with a spectrophotometer. The cDNA library of influenza RNA was prepared using the QIAseq FX single cell RNA library kit (Qiagen, Hilden, Germany). The library concentration was measured by LightCycler qPCR (Roche, Basel, Switzerland), and the library size was checked using TapeStation HS D5000 Screen Tape (Agilent Biotechnologies).

For cluster generation, the library was loaded into a flow cell in which fragments were captured on a lawn of surface-bound oligoes complementary to the library adapters. Each fragment was then amplified into distinct, clonal clusters through bridge amplification. When the cluster generation was complete, the templates were used for sequencing.

Illumina SBS technology utilizes a proprietary reversible terminator-based method that detects single bases as they are incorporated into DNA template strands. As all 4 reversible, terminator-bound dNTPs are present during each sequencing cycle, natural competition minimizes incorporation bias and greatly reduces raw error rates compared to other technologies. The result is highly accurate base-by-base sequencing that virtually eliminates sequence context-specific errors, even within repetitive sequence regions and homopolymers.

Sequencing data was converted into raw data for analysis.

### 2.5. Phylogenetic Analysis

All reference sequences used in this study were obtained from both the NCBI Influenza Virus Resource (https://www.ncbi.nlm.nih.gov/genbank/) and GISAID (http://www.gisaid.org). Homology analysis of nucleic acids was performed on the NCBI and/or GISAID website with BLAST. The phylogenetic relationships of the aligned sequences for eight gene segments were determined by alignment using MUSCLE and edited with Molecular Evolutionary Genetics Analysis Version 6.0 (MEGA6) [30]. Phylogenetic tree investigation was performed by the neighbor-joining method with 1000 bootstrap replicates by MEGA6 software. The following eight gene segments were aligned: polymerase basic 2 (PB2): 2313 nucleotide (nt), polymerase basic 1 (PB1): 2288 nt, polymerase acidic (PA): 2199 nt, HA: 1705 nt, nucleoprotein (NP): 1532 nt, NA: 1425 nt, matrix (M): 1001 nt, and nonstructural (NS): 864 nt.

### 2.6. Measurement of 50% Tissue Culture Infectious Dose (TCID_50_) and 50% Egg Infectious Dose (EID_50_)

The TCID_50_ (50% tissue culture infectious dose) titers were determined by the enzyme-linked immunosorbent assay (ELISA) as previously reported [31]. Briefly, Madin-Darby Canine Kidney (MDCK) cells were grown on flat-bottom 96-well plates and washed with phosphate-buffered saline and then the cells were inoculated with serial 10-fold dilutions of virus samples. Inoculated cells were incubated at 37 °C, 5% CO_2_ and the TCID_50_ titers were determined for 3 days and calculated by Reed and Muench [32]. To evaluate the growth kinetics of A/Mandarin duck/South Korea/KNU18-12/2018 (H11N9) and A/California/04-005-MA/2009(H1N1) (abbreviation: A/California/04/2009 (H1N1)) virus in vitro, the MDCK cell was infected with the virus at a multiplicity of infection (MOI) of 0.001. After a 1 hour incubation, the virus inoculum was removed, the cells were washed 3 times with PBS and cultured in a media infection medium containing 1 μg/mL l-1-tosylamide-2-phenylethyl chloromethyl ketonetreated trypsin. Supernatants were collected at 12, 24, 36, 48, 60, and 72 hours postinfection (hpi). The virus titer of each supernatant was determined by TCID_50_ assay in MDCK cells [33].

To measure EID_50_, serial 10-fold dilutions of the virus were prepared and 100 μL of each dilution was inoculated into the chorio-allantoic cavities of 10-day-old SPF-embryonated chicken eggs. The eggs were incubated at 37 °C for 3 days. Five eggs were infected with each virus dilution. Harvested allantoic fluid was tested for hemagglutination (HA) activity [34] and EID_50_ [33]. 

### 2.7. Animal Infection

To determine the pathogenic potential of the new isolate in mammals, 10^5^ EID50/50 μL of virus was intranasally inoculated to six-week-old female BALB/c mice (*n* = 5), which were purchased from Orient (Seongnam, Gyeonggi, Korea). Anesthesia was conducted with 1% isoflurane following the manufacture’s instruction (Hana Pharmacy, Hwasung, Korea) under the guidelines of Vertebrate animal research, the University of Iowa [35]. The body weight and survival rate of mice were observed for 14 days. At day 3, 6, and 14 days postinfection, mice (*n* = 3) were euthanized and their lungs were harvested. This tissue was homogenized, and the viral titers of homogenate supernatant were determined by TCID_50_ [31]. This study was approved by the Animal Ethics Committee of Wonkwang University (WKU19-64), and all methods were carried out in accordance with relevant guidelines and regulations.

### 2.8. Statistics

The mean, SD, and Student’s *t*-test were conducted using GraphPad Prism. Results were presented as the mean ± SD. A value of *P* < 0.05 was considered significant. 

## 3. Results

### 3.1. Genetic Characterization of Novel Avian Influenza A (H11N9) in 2018

Fecal samples from waterfowls were collected from Gyeonggi, South Korea (37°51′00.93″, 126°46′21.95″) on July 9 2018 (Figure S1). The host was identified as Mandarin duck (*Aix galericulata*) (Figures S1 and S2). The sequence of cytochrome c oxidase subunit I (*COI*) gene (509 bp) is shown in Table S1 and was found very close to the habitat in which avian influenza A virus was introduced into Korea from North America by wild birds [36]. Resident information for Mandarin ducks was obtained in March and October in 2018, as confirmed by the National Institute of Biological Resources in Korea (Figure S3). 

The virus was inoculated in embryonated chicken eggs and then an HA assay and specific PCR amplification were conducted to determine the subtype and all sequences of the AIV by NGS using total RNA (GnCBio) (Table S2). The sequence was identified as A/Mandarin duck/South Korea/KNU18-12/2018(H11N9). The GenBank accession numbers of the eight gene segments are shown in Table S3.

Surface genes (HA and NA) and two internal genes (NS and NP) of A/Mandarin duck/South Korea/KNU18-12/2018(H11N9) were closely related to isolates from Japan, whereas only the M gene was related to newly isolated virus from wild waterfowl in Korea (2018). In contrast, other internal genes (PB1 and PA) were similar to those of isolated strains from China, whereas PB2 was correlated with a Russian isolate. Specifically, our A/Mandarin duck/South Korea/KNU18-12/2018(H11N9) showed the highest identity with both the HA and NA genes of the A/duck/Kagoshima/KU57/2014 (H11N9) strain, with 97.70% and 98.00% nucleotide identities, respectively. The M gene was most closely related to A/wild waterfowl/Korea/F7-18/2018 (H4N8), with 99.80% identity. Both the PA and PB1 genes were closely related to the H5N3 strains in China, with identities of 99.21% and 99.08%, respectively. The remaining genes in our isolate (NP, PB2, and NS) showed a close relationship with A/duck/Aichi/231003/2016 (H8N4), A/mallard/Khabarovsk/241/2017 (H10N6), and A/avian/Japan/8KI0162/2008 (H3N8), respectively (Table 1). In contrast, A/waterfowl/Korea/S353/2016 (H11N9) showed lower genetic similarity with A/Mandarin duck/South Korea/KNU18-12/2018(H11N9) in all gene segments.

**Figure 1:**
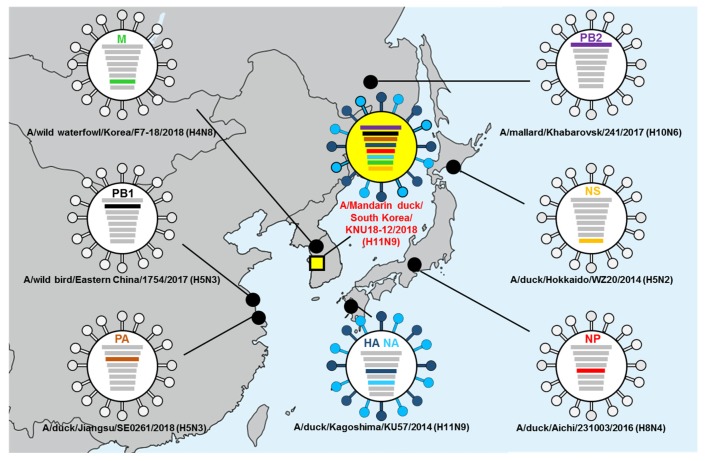
Location of putative origin of genomic compositions of the A/Mandarin duck/South Korea/KNU18-12/2018(H11N9).

The spatial locations of all virus strains, including the gene related to our H11N9 strain, are illustrated in Figure 1.

**Figure 2:**
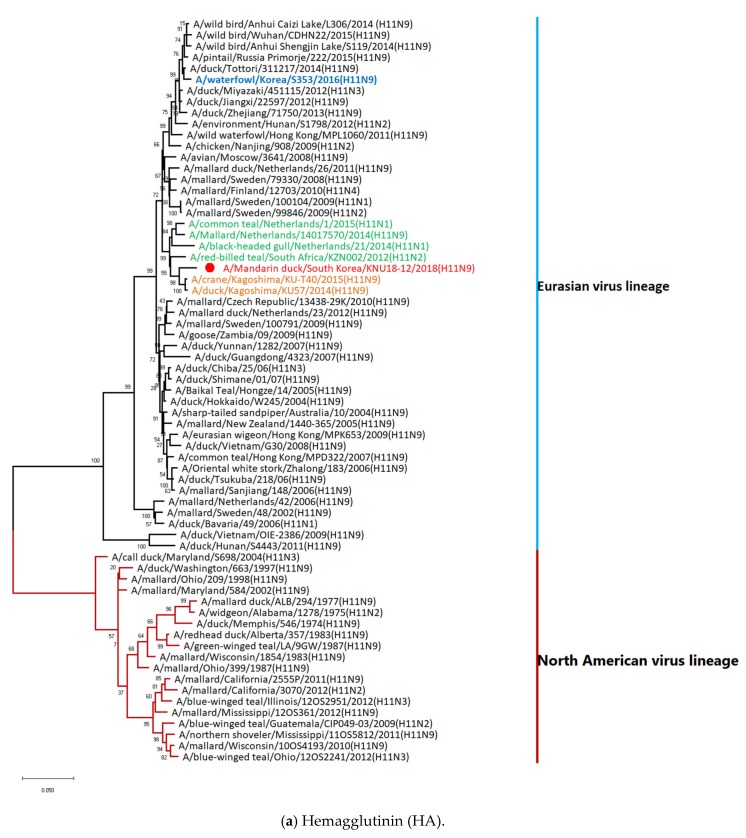

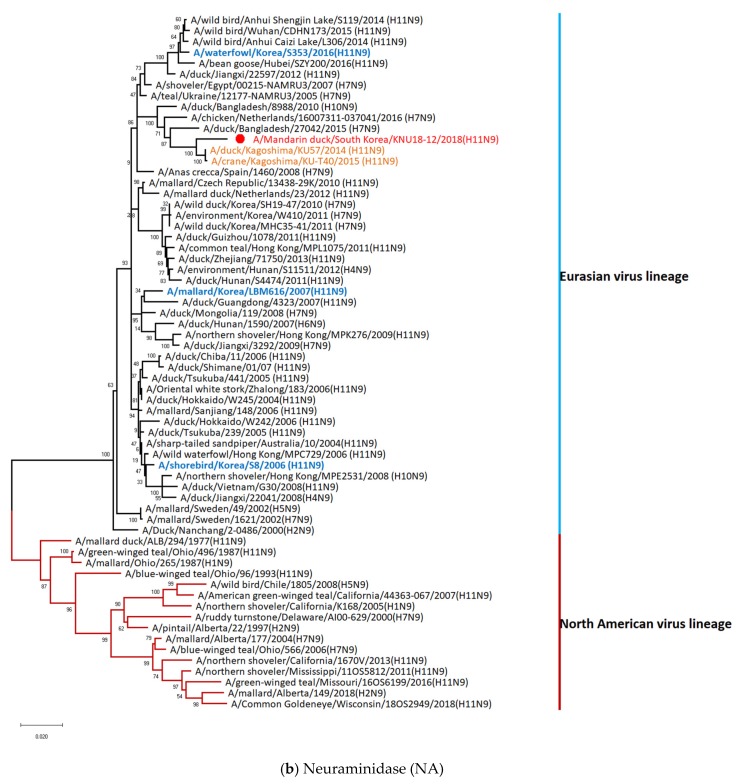

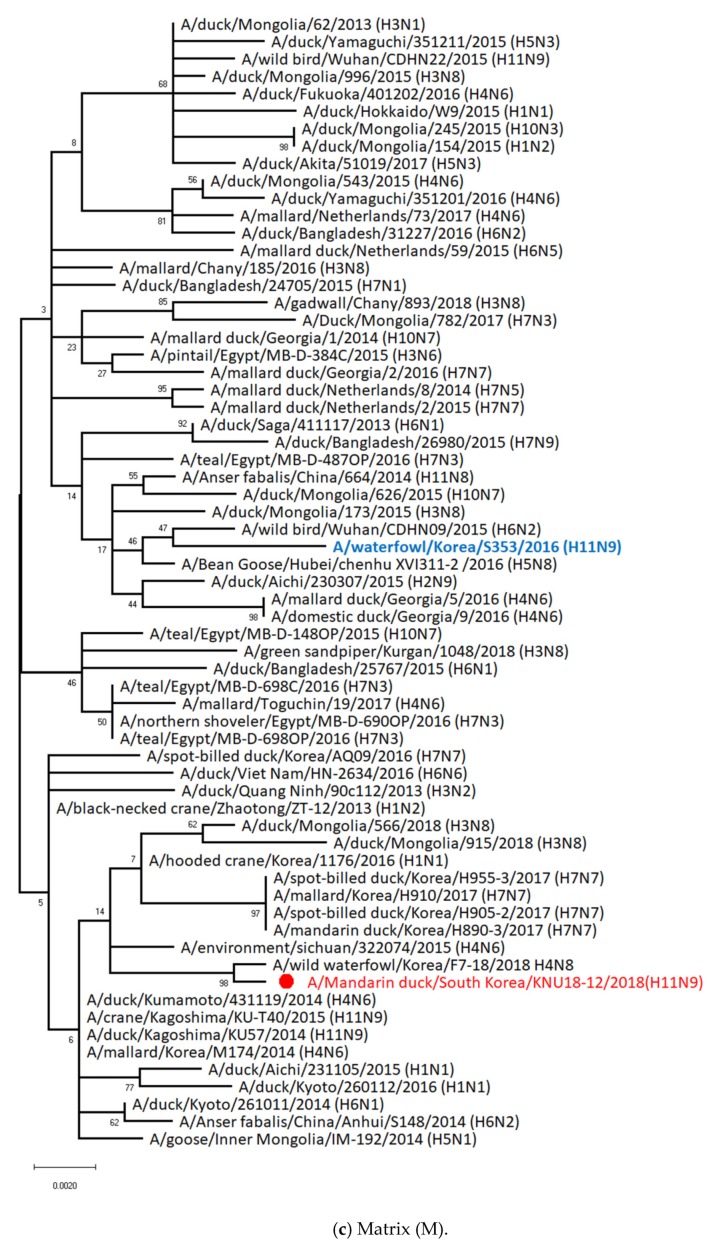

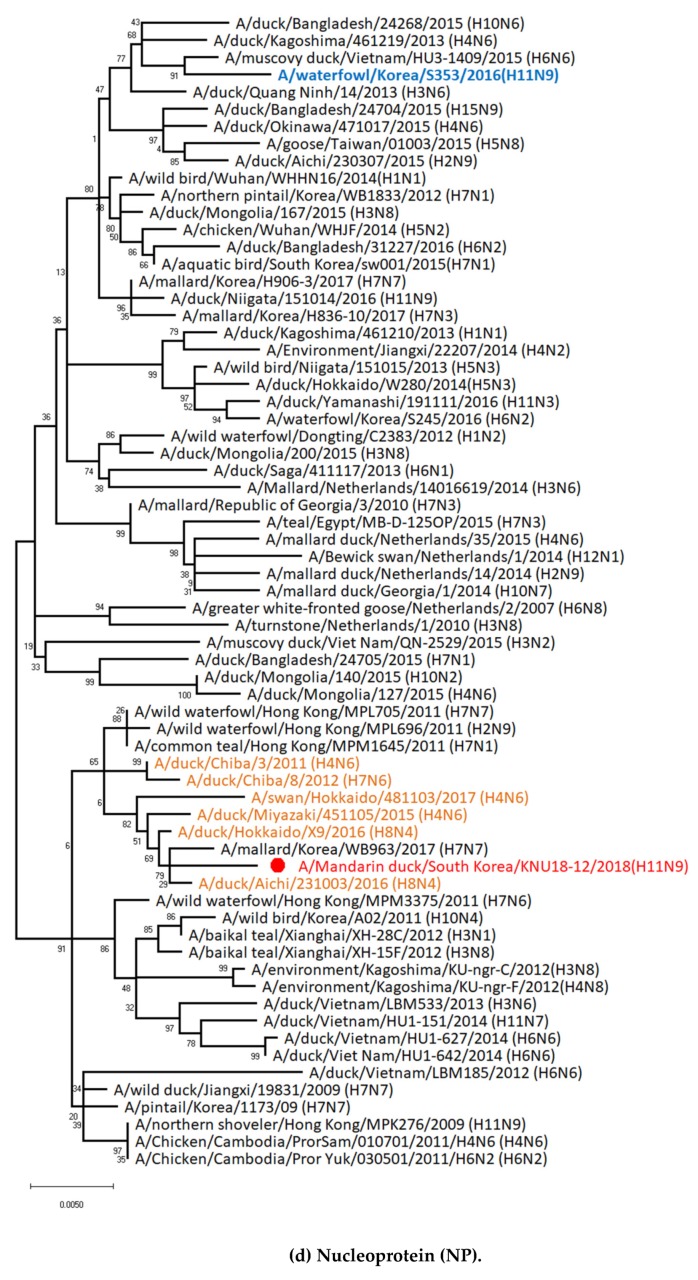

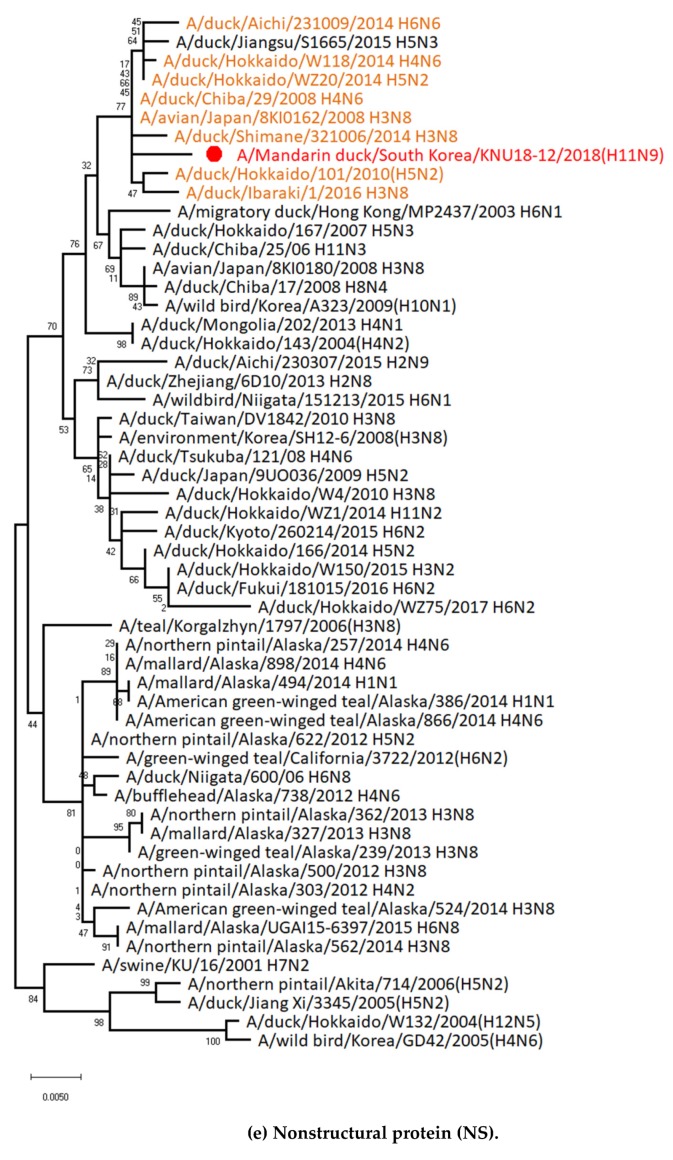

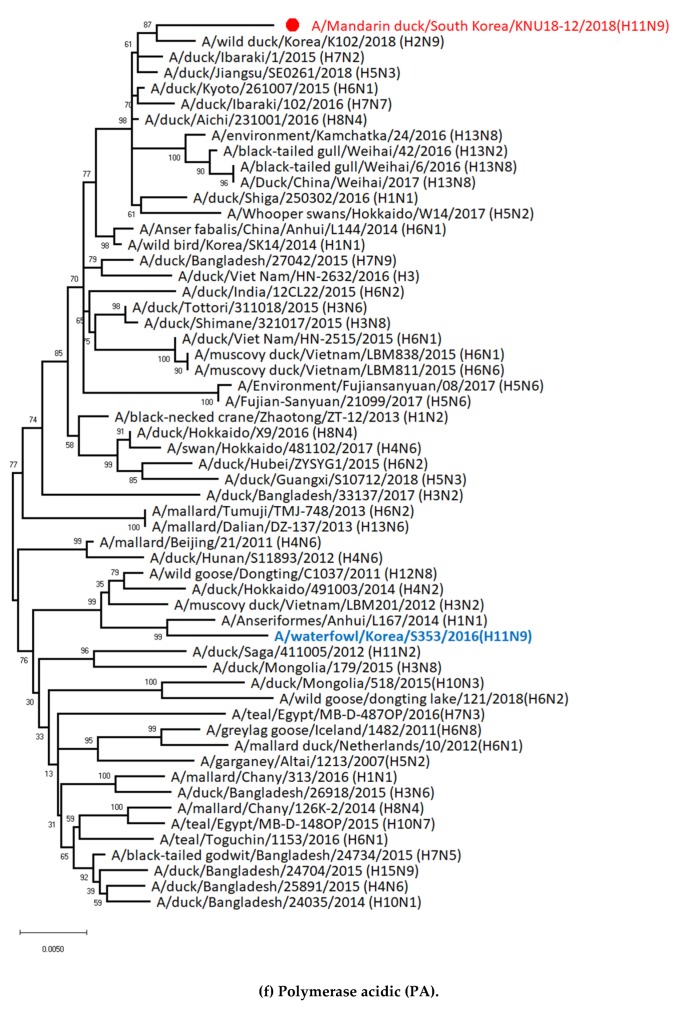

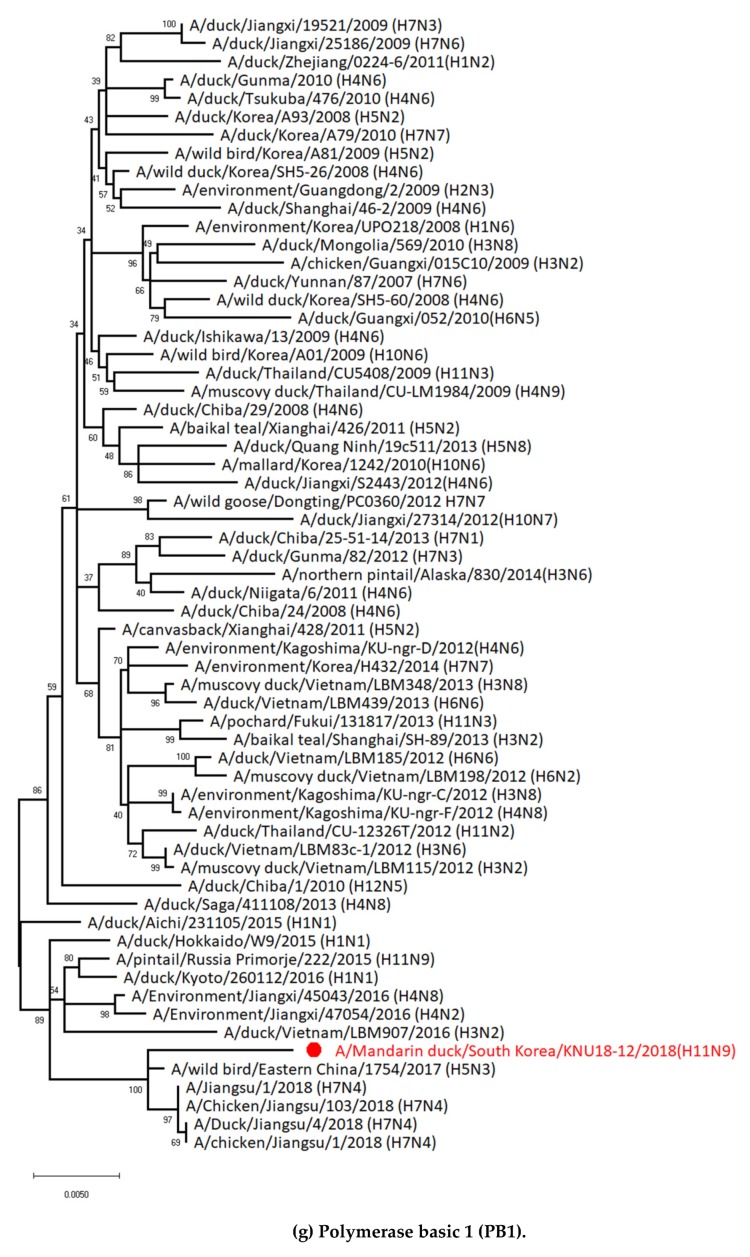

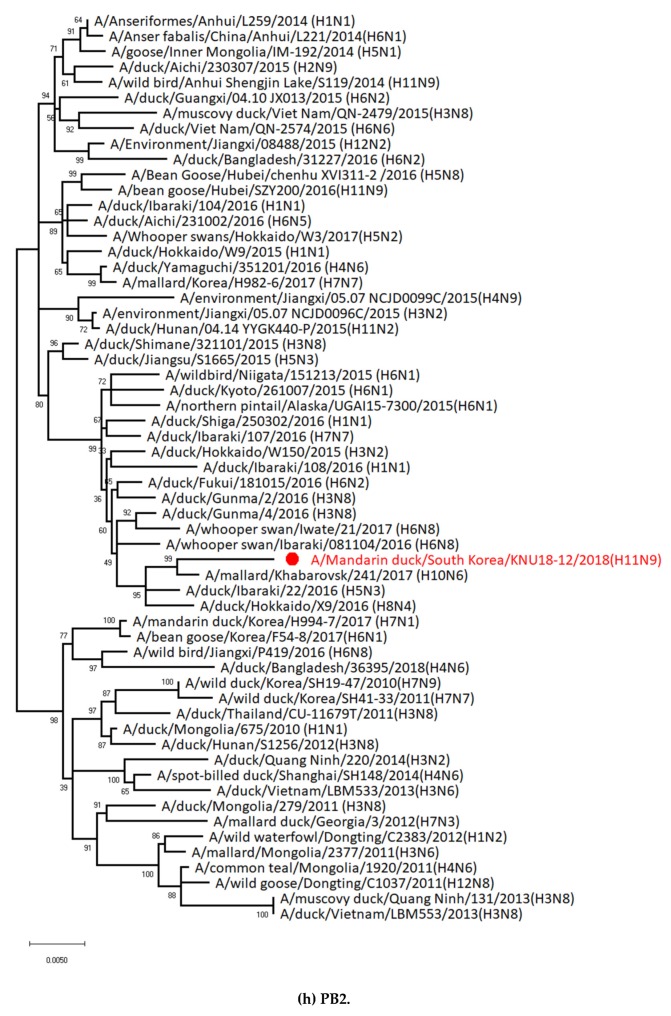
Phylogenetic tree based on nucleotide sequences of HA (**a**), NA (**b**), M (**c**), NP (**d**), NS (**e**), PA (**f**), PB1 (**g**), and PB2 (**h**). The tree was generated by the neighbor-joining method with MEGA 6.0 software using bootstrap replication (1000 bootstraps). Our isolate (A/Mandarin duck/South Korea/KNU18-12/2018(H11N9)) is indicated by a red color and A/waterfowl/Korea/S353/2016 (H11N9), a previous H11N9 isolate in Korea, is indicated in blue. The Japan virus and origin strain of the Japan strain are shown in orange and green, respectively.

Phylogenetic analysis for the eight genes of AIV A/Mandarin duck/South Korea/KNU18-12/2018(H11N9) was performed to assess their genetic relationships with those of domestic poultry and wild birds in Korea and neighboring countries using data from both the NCBI and GISAID. The results revealed that eight genes (PB2, PB1, PA, HA, NP, NA, M, and NS) in our H11N9 strain were distributed in Eurasian lineages (Figure 2). Particularly, both the HA and NA genes in this virus were closely related to those of A/duck/Kagoshima/KU57/2014 (H11N9) and A/crane/Kagoshima/KU-T40/2015 (H11N9) rather than with the previously reported H11N9 in Korea (2016), according to genetic homology analysis using BLAST. The NP and NS genes may have been simultaneously introduced into Korea by Japanese strains rather than from other countries. Based on the phylogenetic tree, the M gene was close to clusters from Japan (2014–2015) and China/Mongolia (2015), although it showed the highest similarity with the Korean 2018 isolate. Additionally, the PB2 gene originated from Japanese clusters in 2016, although it was mostly related to Russian strains. The PA and PB1 genes have been introduced from the Chinese cluster in 2018. 

### 3.2. Molecular Characterization of H11N9 Isolate

Highly pathogenic AIVs are characterized by a series of basic amino acids at the HA cleavage site which enable the systemic spread of the virus. Five amino acids in the HA protein (position 138, 190, 225, 226, and 228; H3 numbering) were adaptive mutation positions allowing the host to change from birds to humans [37]. The HA of this isolate was compared to a strain isolated from swine in Korea (A/swine/KU/2/2001 (H11N6)), a strain isolated from waterfowl in Korea (A/waterfowl/Korea/S353/2016 (H11N9)), a strain isolated from duck in Japan (A/duck/Kagoshima/KU57/2014) which was closely related to our H11N9 strain, and three H11N9 strains isolated from avian (A/duck/Vietnam/OIE-2386/2009 (H11N9), A/goose/Zambia/09/2009 (H11N9), and A/duck/Vietnam/LBM81/2012 (H11N9)), which are closely related to the genesis of human H7N9 virus [38].

The HA cleavage site, mutation sites for efficient binding to avian-like α-2,3-linked sialic acid receptors, and the NA stalk region indicate a low-pathogenicity avian influenza (LPAI) (Table 1) [37].

Furthermore, the NA gene in this isolate contained arginine at positions 152 and 292 and histidine at position 274, indicating susceptibility to the NA inhibitors oseltamivir and zanamivir, which is similar to other H11N9 strains in and near Korea as well as A/swine/KU/2/2001(H11N6) [39].

Mutations in internal genes which may increase replication efficiency as well as the virulence of a virus in different hosts indicate that H11N9 is virulent in avian and mammals as observed for previous H11N9 strains (Table 2).

### 3.3. Replication of H11N9 in Mammalian Cells

As the genetic information implied that novel reassortment of H11N9 could increase replication efficiency, the viral replication of H11N9 was tested in vitro.

Analysis of the TCID_50_, plaque assay, and/or quantitative reverse transcription PCR with mathematical modelling are powerful approaches for performing detailed characterization of viral replication in vitro [61].

**Figure 3:**
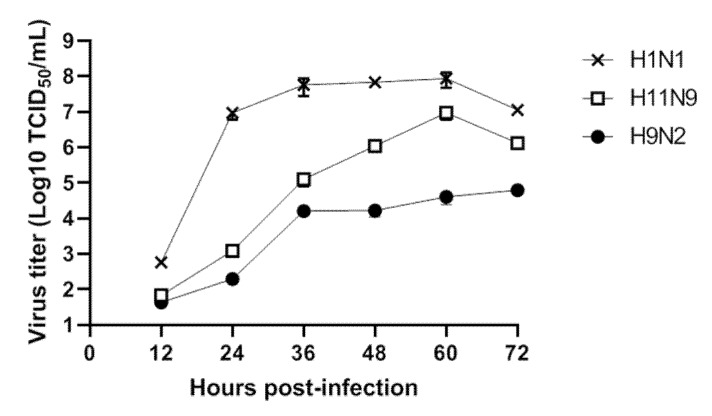
In vitro growth properties of H11N9 in Madin-Darby Canine Kidney (MDCK) cells. Virus titers were determined by using the 50% tissue culture infectious dose (TCID_50_) assay. Cell monolayers were infected with viruses at an MOI of 0.001 and TCID_50_/mL of supernants was collected at different time for 72 h. At every 12 hours, the TCID_50_ of supernatant of cells was measured by ELISA with anti-influenza nucleoprotein to determine the infected cells. The virus titers are means ± standard deviations (SD) (*n* = 3).

Monitoring of influenza virus shedding is important in the investigation of virus adaptation to mammalian host. The human-origin virus, A/California/04/2009 (H1N1), which was donated from Korea National Institute of Health (Chungju, Korea), was used as a control. DCK cells were infected with virus (MOI = 0.001). At every 12 hours, supernatants were collected and conducted for a TCID_50_ assay using the ELISA method to determine the virus-positive cells as previously described [31]. The H1N1 replicated more efficiently in MDCK cells, as compared with A/Mandarin duck/South Korea/KNU18-12/2018(H11N9) (Figure 3). Chicken-origin virus (A/chicken/Korea/KNUSWR09/2009 (H9N2)) [62] showed the lowest titer at each investigated time. Raw data of TCID_50_ assay are shown in Figure S4.

### 3.4. Pathogenicity in Mice

**Figure 4:**
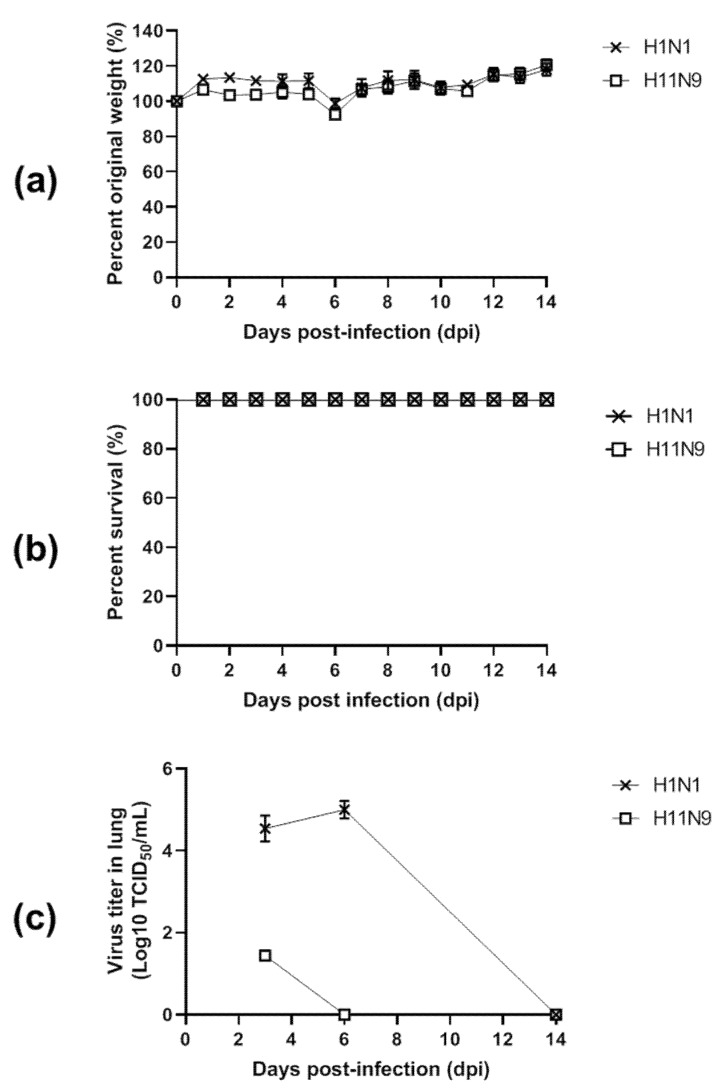
Pathogenicity of H11N9 virus in vivo. Balb/c were intranasally challenged with 10^5^ EID_50_ of virus. H1N1 was used as a control virus. Mice weight (**a**) and survival rate (**b**) were observed for 14 dpi. Body weight was presented as % of those of original mice (*n* = 5). Mean virus titers in the lungs of mice (*n* = 3) were measured at 3, 6, and 14 dpi (**c**).

To determine the pathogenic potential of the new isolate in mammals, 10^5^ EID_50_/50 μL of each virus was used to inoculate five 6-week-old female BALB/c mice. The infected mice displayed no severe clinical signs, including ruffled fur, depression, labored breathing and severe weight loss during 14 days postinfection (dpi) (Figure 4a). All mice infected with control (H1N1) and challenge (H11N9) survived within 14 dpi, showing no statistical significance in the survival rate (Figure 4b). The kinetics of replication of H11N9 in mice is displayed in Figure 4c. H11N9 replicated in lung at a lower titer (1.43 ± 0.12 log10 TICD_50_/mL at 3 dpi and absent in lung at 6 and 14 dpi while H1N1 maintained a comparatively high titer at 6 dpi (4.94 ± 0.18 log10 TICD_50_/mL) (Figure S5). 

### 3.5. Hypothesis of Reassortment Event of A/Mandarin Duck/South Korea/KNU18-12/2018(H11N9)

**Figure 5:**
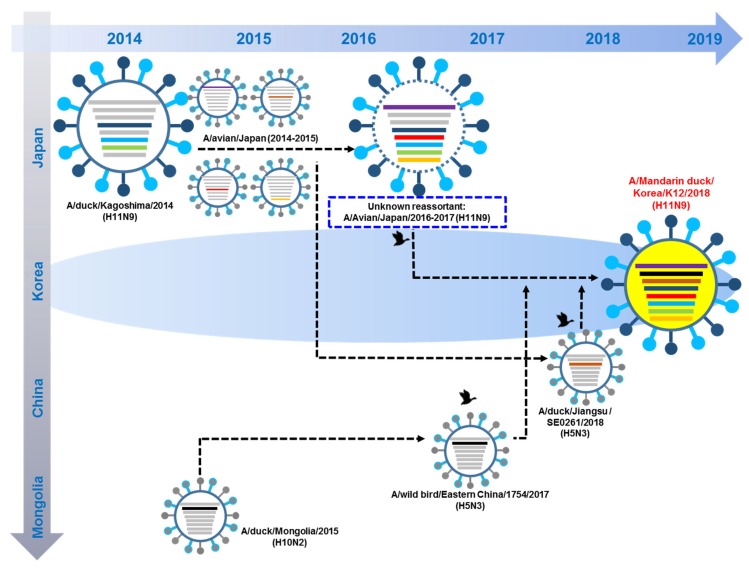
Original reassortment events of novel avian influenza A/Mandarin duck/South Korea/KNU18-12/2018(H11N9). The 8 gene segments (from top to bottom) in each virus are polymerase basic 2 (-), polymerase basic 1(-), polymerase acidic (-), hemagglutinin (-), nucleoprotein (-), neuraminidase (-), matrix (-), and nonstructural (-). Each virus diagram indicates a separate virus.

In 2014–2015, A/duck/Kagoshima virus was reassorted with subtype H11N9 viruses from Japan to produce an unknown A/Avian/ Kagoshima/2017(H11N9) with additional reassortment with other subtypes in 2015–2017. Until late 2017, unknown A/ Avian/ Kagoshima/2017(H11N9) circulated in eastern China and South Korea. In 2018, unknown A/ Avian/ Kagoshima/2017(H11N9) reassorted with PB1 of A/wild bird/Eastern China/1754/2017(H5N3) and PA of A/duck/Jiangsu/SE0261/2018(H5N3) to generate A/Mandarin duck/South Korea/KNU18-12/2018(H11N9) (Figure 5). 

## 4. Discussion

Reassortment among different AIVs is the main mechanism involved in the generation of new types, which may increase mammalian transmission and threaten public health [63]. In Korea, several outbreaks of HPAI have occurred since 2003 [64], which were mostly diffuse among wild birds at breeding or staging sites, such as central Asia and Siberia, after which these viruses then disseminated into eastern Asia. There may be some degree of temporal-spatial segregation across Eurasian wild bird populations, resulting in the maintenance of distinct variants and, therefore, heterogeneity in emerging strains in different geographic regions [3].

Compared to imported strains from Russia–Mongolia-China to Korea, there are a few reports of genetic similarity between Japan- and Korea-derived AIVs [65]; mountain Hawk Eagles and Mandarin ducks showed a close ancestor for the H5N1 and H5N6 strains in Japan and Korea, respectively [66]. The H5 and H7 subtypes are highly pathogenic AIVs; however, low-pathogenic avian viruses can function as donors or precursors of highly pathogenic strains [63,67,68]. No studies have compared the genetic similarity of LPAI between Japan and Korea. To predict outbreaks of high-pathogenicity avian influenza (HPAI), stringent surveillance and analysis of gene diversity of LPAIs are needed.

H11N9 has been found in wild birds in Brazil, Europe, and Japan [14,17,19,21,22,69,70]. Japan is an overwintering site of endangered cranes (hooded cranes and white-naped cranes) and many other migratory birds (including wild ducks), which are considered as carriers of AIVs [70]. For the 2014–2015 winter season, all genes of the two H11N9 isolates were found to be related to AIVs belonging to the Eurasian virus lineage. Notably, the H11 HA and N9 NA genes showed high sequence similarity to the corresponding genes of isolates from wild birds in South Africa and Spain, respectively, and did not cluster in the major groups with recent wild-bird isolates from East Asia, suggesting that dynamic movements of wild birds in Japan occur in the Izumi plain. Previously, H11N9 in China was detected from avian birds in 2006–2015 and in environmental samples from live poultry markets in China in 2016 [69]. This indicates that Chinese H11N9 obtained the potential for transmission from avians to poultry. 

All three strains isolated from Brazil consistently clustered together within evolutionary lineages of AIVs that had been previously described in aquatic birds in North America. Particularly, the H11N9 isolates were remarkably closely related to AIV strains from shorebirds sampled at the Delaware Bay region, on the Northeastern coast of the USA, more than 5000 km away from where the isolates were retrieved. Additionally, there was evidence of genetic similarity to AIV strains from ducks and teals from the interior USA and Canada [17]. Our isolate was not closely related to the Brazilian strains, showing genetic homology below 90% (Table S4). 

The H11N9 strain analyzed in this study consistently clustered together within evolutionary branches of AIVs previously described in migratory birds in Japan. Six internal genes were closely related to AIV strains identified in migratory birds in Japan. The only exceptions were the PB1 and PA sequences, which were closely related to the Chinese AIV strains. PB1 clustered into Chinese strains between 2017 and 2018. Although substantial genetic similarity of PA was observed in A/duck/Jiangsu/SE0261/2018 (isolated in January 2018), it clustered with Japanese strains between 2015 and 2017 in phylogenetic analysis.

According to the phylogenetic tree of HA and NA, the newly identified H11N9 clustered with the same strains as the most genetically similar strain (A/A/duck/Kagoshima/KU57/2014 (H11N9) rather than with Korean H11N9 found in 2016 and 2014–2015; dead spot-billed ducks carried two AIVs of the H11N9 subtype in the Izumi plain [70]. As spot-billed ducks are widely distributed in the west and along the southern coastline in Korea, Japan, China, and Russia [71], this bird may be one of the hosts that migrated to Korea with the H11N9 reassortant in 2016–2017 (Figure 3).

Furthermore, our H11N9 had a lower in vitro titer in MDCK cells than A/California/04/2009 (H1N1), supporting the LPAI characterization of H11N9. Although newly found H11N9 in 2018 exhibited low pathogenicity in mice, maintaining a database for H11N9 through careful surveillance is important because of its wide host tropisms (swine pig as host) and relatedness to H11N9 to contribute to the genesis of HPAI H7N9. 

From the in vivo study, the presence of H11N9 was observed in lungs of mice at 3 dpi but it was not detected at 6 dpi, while A/California/04/2009 (H1N1) was observed in lung of mice by 6 dpi, indicating the lower mouse adaptation of H11N9 than A/California/04/2009 (H1N1). A/California/04/2009 (H1N1)-infected mice survived up to 14 dpi without change of body weight, which corresponds to other previous reports [72,73]. It may be caused by the inoculation of a relatively low titer (< 10^4.67^ TCID_50_/mL) of A/California/04/2009 (H1N1) virus to mice in this study (Table S5). Mouse adaption of A/California/04/2009 (H1N1) can be increased by a total of nine passages in mice with nine new mutations [73] and A/California/04/2009 (H1N1) used in this study showed only three out of nine amino sites, supporting that A/California/04/2009 (H1N1) in this study was not a mouse-adaptive strain (Table S6). 

Satellite-tracking data supported that the crane used an eastern migration route from the Izumi plain in Japan followed the west coast of South Korea in the spring, moved to China for breeding in summer, and moved down to South Korea in autumn, after which it returned to the Izumi plain in Japan in winter in 2014–2016 [74].

Interestingly, not only the H11 HA and N9 NA genes but also our isolate showed high sequence similarity to the corresponding genes of isolates from wild birds in South Africa and Spain, respectively, rather than those from East Asia, supporting that our isolate is related to the Japanese strains. 

The closest strains to A/Mandarin duck/South Korea/KNU18-12/2018(H11N9) HA were A/crane/Kagoshima/KU-T40/2015(H11N9) and A/duck/Kagoshima/KU57/2014(H11N9), showing HA from South Africa. These strains were closely related to A/red-billed teal/South Africa/KZN002/2012 (H11N2). The closest strain to A/Mandarin duck/South Korea/KNU18-12/2018 (H11N9) NA was A/crane/Kagoshima/KU-T40/2015 (H11N9) and A/duck/Kagoshima/KU57/2014 (H11N9), which originated from A/Anas crecca/Spain/1460/2008 H7N9. These results correspond to the previously identified H11N9 in the Izumi plain in Kagoshima, Japan [70]. 

Wild aquatic birds such as dabbling ducks, gulls, and other shorebirds are considered as natural reservoirs for AIVs [75]. H11N9 has been widely detected in mallard ducks, sharp-tailed sandpipers, and pekin ducks as hosts [6,76], and infection with H11N2 subtype AIVs was confirmed in Adélie penguins in Antarctica [77]. A recent study identified mandarin ducks as hosts for H11N9 in 2008 [20]. Mandarin ducks are distributed in East Asia and Europe and are year-round residents in South Korea and Japan; others populations migrate to China [78]. Although they are widely distributed in East Asia, Mandarin ducks are less susceptible to AIVs compared to other wild ducks. 

H5 subtypes have mainly been found in Mandarin ducks. However, the susceptibility of Mandarin ducks to AIVs differs even for highly pathogenic AIVs depending on the genotypes. Mandarin ducks have not been shown to be susceptible to infection with Clade 2.3.4.4 Group B H5N8 and Group C H5N6 viruses [79]. In contrast, this duck carried the HPAI reassortant avian influenza A (H5N6) group C virus in South Korea, 2016 (MD/KR/2016), and the HA gene of the MD/KR/2016 virus belonged to and clustered with the H5N6 subtype viruses isolated from humans, cats, and the environment in Guangdong, China during 2014–2015 [80]. H6N5 is rarely detected in Mandarin ducks [36]. 

Recently, A/duck/Jiangsu/SE0261/2018 (H5N3) was isolated from domestic ducks in a poultry market in Jiangsu Province in the spring of 2018 [81].

Interestingly, A/Mandarin duck/South Korea/KNU18-12/2018(H11N9) showed the highest PA gene similarity with A/duck/Jiangsu/SE0261/2018 (H5N3). Similarly, the other different subtype isolate (H2N9) [82] isolated in this surveillance showed the highest PA genetic similarity to A/duck/Jiangsu/SE0261/2018 (H5N3). As two different subtypes found in Korea in 2018 possessed the same strain, A/duck/Jiangsu/SE0261/2018 (H5N3), in terms of PA genetic similarity, we suggest that A/duck/Jiangsu/SE0261/2018 (H5N3) moved to Korea and reassorted to produce Korean H2N9 and H11N9 by donating the PA gene (Figure S6). 

## 5. Conclusions

We isolated a novel H11N9 in 2018 which was genetically different from previously reported Korean H11N9 in 2016. Although this research was based on the analysis of limited genetic diversity, the most likely route of infection of A/Mandarin duck/South Korea/KNU18-12/2018(H11N9) may be a reassortant virus shed by wild migratory birds in Japan and China to Korea.

## Figures and Tables

**Table 1:** Comparison of amino acid in hemagglutinin (HA) receptor-binding site and neuraminidase gene segment of Swine and Avian H11 influenza virus in Korea and near Korea.

Virus Strain.	HA Receptor Binding Residues (H3 Numbering).						NA			
	Cleavage Sites	A138S	E190D	G225D	Q226L	G228S	69–73 (QISNT)	152	274	292
A/Mandarin duck/South Korea/KNU18-12/2018(H11N9)	PAIASR↓GLF	A	E	G	Q	G	No deletion	R	H	R
A/waterfowl/Korea/S353/2016 (H11N9)	PAIASR↓GLF	A	E	G	Q	G	No deletion	R	H	R
A/duck/Vietnam/OIE-2386/2009 (H11N9)	PAIASR↓GLF	A	E	G	Q	G	No deletion	R	H	R
A/goose/Zambia/09/2009 (H11N9)	PAIASR↓GLF	A	E	G	Q	G	No deletion	R	H	R
A/duck/Vietnam/LBM81/2012 (H11N9)	PAIASR↓GLF	A	E	G	Q	G	No deletion	R	H	R
A/duck/Kagoshima/KU57/2014 (H11N9)	PAIASR↓GLF	A	E	G	Q	G	No deletion	R	H	R
A/swine/KU/2/2001 (H11N6)	PAIASR↓GLF	A	E	G	Q	G	Deletion	R	H	R

**Table 2:** Identification of amino acids of the influenza A(H11N9) viruses involved in enhancing antiviral drug resistance and causing pathogenesis in mammals.

Viral Protein.	Amino Acid	K/2018 ^a^	K /2016 ^b^	Za/2009 ^c^	Vi/2012 ^d^	Sh/2013 ^e^	An/2013 ^f^	Ka /2014 ^g^	Comments	Reference
PB2	E627K	E	E	E	E	K	K	E	Mammalian host adaptation	[39,40,41,42,43]
	D701N	D	D	D	D	D	D	D	Increase polymerase activity and viral replication in mammalian cells	[39,40,41,42,43]
	L89V	V	V	V	V	V	V	V	Enhanced polymerase activity, Increased virulence in mice	[44,45]
	G309D	D	D	D	D	D	D	D	Enhanced polymerase activity, Increased virulence in mice	[45]
	T339K	K	K	K	K	K	K	K	Enhanced polymerase activity, Increased virulence in mice	[45]
PA	V100A	V	V	V	V	A	A	V	Contributed to the virulence and mammalian adaptation	[41,42,46]
	K356R	K	K	K	K	R	R	K	Contributed to the virulence and mammalian adaptation	[47]
	S409N	S	S	S	S	N	N	S	Contributed to the virulence and mammalian adaptation	[41,46]
	A515T	T	T	T	T	T	T	T	Increased polymerase activity, Increased virulence in mammals and birds	[48,49]
PB1	H436Y	Y	Y	Y	Y	Y	Y	Y	Increased polymerase activity and virulence in mallards, ferrets and mice	[49]
NS1	P/A42S	A	S	S	S	S	S	S	Increased virulence in mice (most avian influenza A viruses encode 42S)	[50,51]
	T/D92E	D	D	D	D	D	D	D	Increased virulence in mammals, Escape of antiviral host response	[50,52,53,54]
M1	N30D	D	D	D	D	D	D	D	Increased virulence in mice (most influenza A viruses encode 30D)	[55,56]
	T215A	A	A	A	A	A	A	A	Increased virulence in mice (most influenza A viruses encode 215A)	[55,56]
M2	L26P	L	L	L	L	L	L	L	Reduced susceptibility to amantadine and rimantadine	[57]
	V27A/I	V	V	V	I	V	V	V	Reduced susceptibility to amantadine and rimantadine	[57]
	A30T	A	A	A	A	A	A	A	Reduced susceptibility to amantadine and rimantadine	[57]
	S31N	S	S	S	S	N	N	S	Reduced susceptibility to amantadine and rimantadine	[57,58,59]
PB1-F2	N66S	N	N	N	N	N	N	S	Increased virulence in mammals	[60]

^a^ K/2018: A/Mandarin duck/South Korea/KNU18-12/2018(H11N9). ^b^ K/2016: A/waterfowl/Korea/S353/2016 (H11N9). ^c^ Za/2009: A/goose/Zambia/09/2009 (H11N9). ^d^ Vi/2012: A/duck/Vietnam/LBM81/2012 (H11N9). ^e^ Sh/2013, A/Shanghai/1/2013 (H7N9). ^f^ An/2013, A/Anhui/1/2013 (H7N9). ^g^ Ka /2014: A/duck/Kagoshima/KU57/2014 (H11N9).

## Supplementary Materials

The following are available online at https://www.mdpi.com/1999-4915/12/2/203/s1, Figure S1. Sampling location of A/Mandarin duck/South Korea/KNU18-12/2018(H11N9). Figure S2. Information of bird species identification. Figure S3. Location map of Mandarin ducks marked with satellite transmitters in the East Asian Flyways, March and October in 2018. Figure S4. Raw ELISA data to conduct TCID50 assay. Figure S5. Raw ELISA data to conduct TCID50 assay to measure virus titer in lung. Figure S6. Close relationship of PA gene of different isolates in Korea in 2018. Table S1. Initial input of barcoding. Table S2. Detailed NGS analysis. Table S3. Homology analysis of each gene of A/Mandarin duck/South Korea/KNU18-12/2018(H11N9). Table S4. Genetic similarity with H11N9 strains of different countries. Table S5. Titer of virus used in mouse study. Table S6. Mouse adaptive mutation site of A/California/04/2009 (H1N1)
