# Supplementary material for: Genetic Characterization of Avian Influenza A (H11N9) Virus Isolated from Mandarin Ducks in South Korea in 2018
Source: Viruses. 2020 Feb 12;12(2):203. doi: 10.3390/v12020203 (PMC7077279; doi:10.3390/v12020203)
Supplement: Supplementary file 1 [file viruses-12-00203-s001.pdf]

Supplementary data

Genetic characterization of avian influenza A (H11N9) virus isolated from Mandarin duck in South Korea in 2018

Hien Thi Toung[1], Minh Ngoc Nguyen[1], Haan Woo Sung[2], Hyun Park[1,§], and Seon-Ju Yeo[1,§]

Fig. S1. Sampling location of A/Mandarin duck/South Korea/KNU18-12/2018(H11N9).

Fig. S2. Information of bird species identification

Table S1. Initial input of barcoding

Fig. S3. Location map of Mandarin ducks marked with satellite transmitters in the East Asian Flyways, March and October in 2018.

Table S2. Detailed NGS analysis

Table S3. Homology analysis of each gene of A/Mandarin duck/South Korea/KNU18-12/2018(H11N9)

Fig. S4. Raw ELISA data to conduct $TCID_{50}$ assay.

Fig. S5. Raw ELISA data to conduct TCID50 assay to measure virus titer in lung

Table S4. Genetic similarity with H11N9 strains of different countries

Table S5. Titer of virus used in mouse study

Table S6. Mouse adaptive mutation site of A/California/04/2009 (H1N1)

Fig. S6. Close relationship of PA gene of different isolates in Korea in 2018.

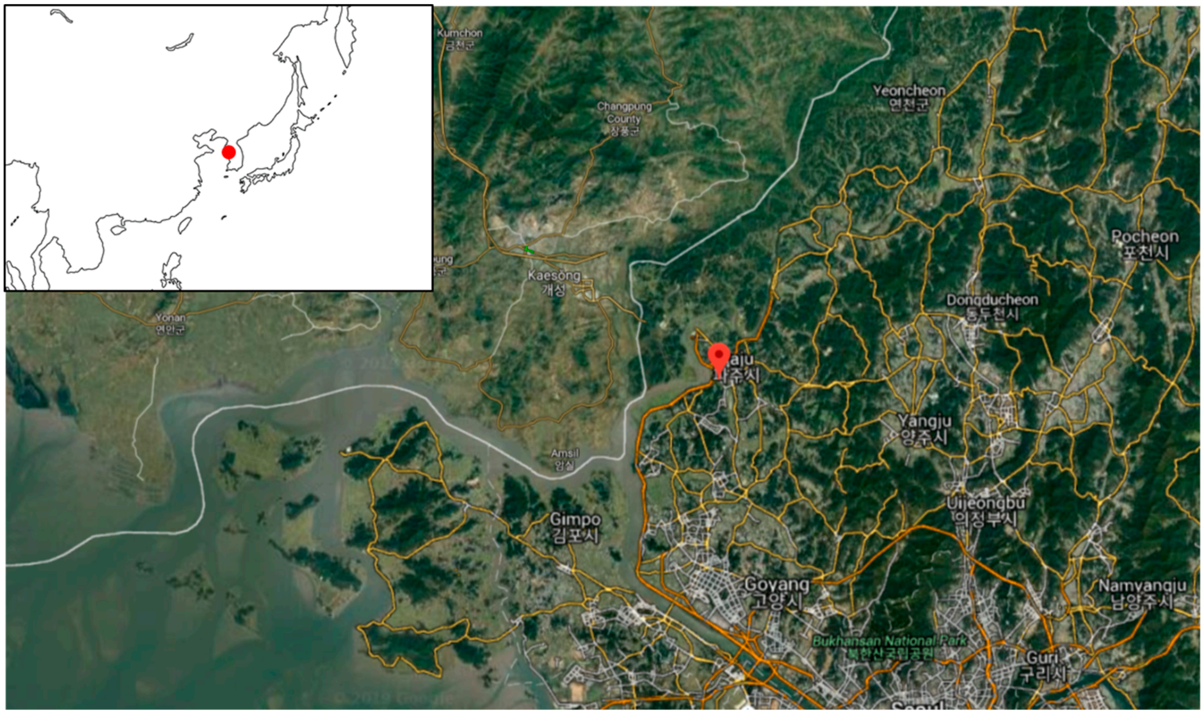

Fig. S1. Sampling location of A/Mandarin duck/South Korea/KNU18-12/2018(H11N9).

BOLD TaxonID Taxonomy Report

Order	Family	Species
Anseriformes[99 individuals][7 species]		
	Anatidae[99 individuals][7 species]	
		Aix galericulata[24 individuals]
		Aix sponsa[9 individuals]
		Anas crecca[59 individuals]
		Cairina moschata[3 individuals]
		Melanitta nigra[1 individuals]
		Somateria fischeri[2 individuals]
		Somateria mollissima[1 individuals]

Fig.

Fig. S2. Information of bird species identification

Table S1. Initial input of barcoding

TAATCTTCTTCATGGTGATACCCATCATAATTGGAGGATTCGGCAATTGACTAGTCC
CCCTAATAATTGGCGCCCCTGACATGGCATTCCCCCGAATGAACAACATAAGCTTCT
GACTCCTTCCACCCTCATTCCTCCTACTGCTCGCCTCATCTACCGTGGAAGCTGGCGC
CGGTACAGGCTGAACCGTGTACCCACCCCTAGCTGGCAACCTAGCCCACGCCGGAG
CCTCAGTAGACCTAGCCATCTTCTCACTCCACTTAGCCGGTGTTTCCTCCATCCTCGG
AGCCATTAACTTCATTACTACGGCCATCAACATAAAACCTCCCGCACTCTCACAATA
CCAAACTCCACTCTTCGTCTGATCCGTCCTAATTACTGCCATCCTACTCCTCCTGTCC
CTCCCCGTTCTTGCCGCTGGCATCACAATGCTACTAACTGACCGAAACCTAAACACC
ACATTCTTCGACCCCGCCGGAGGAGGAGACCCAATCCTGTATCAACACCTA

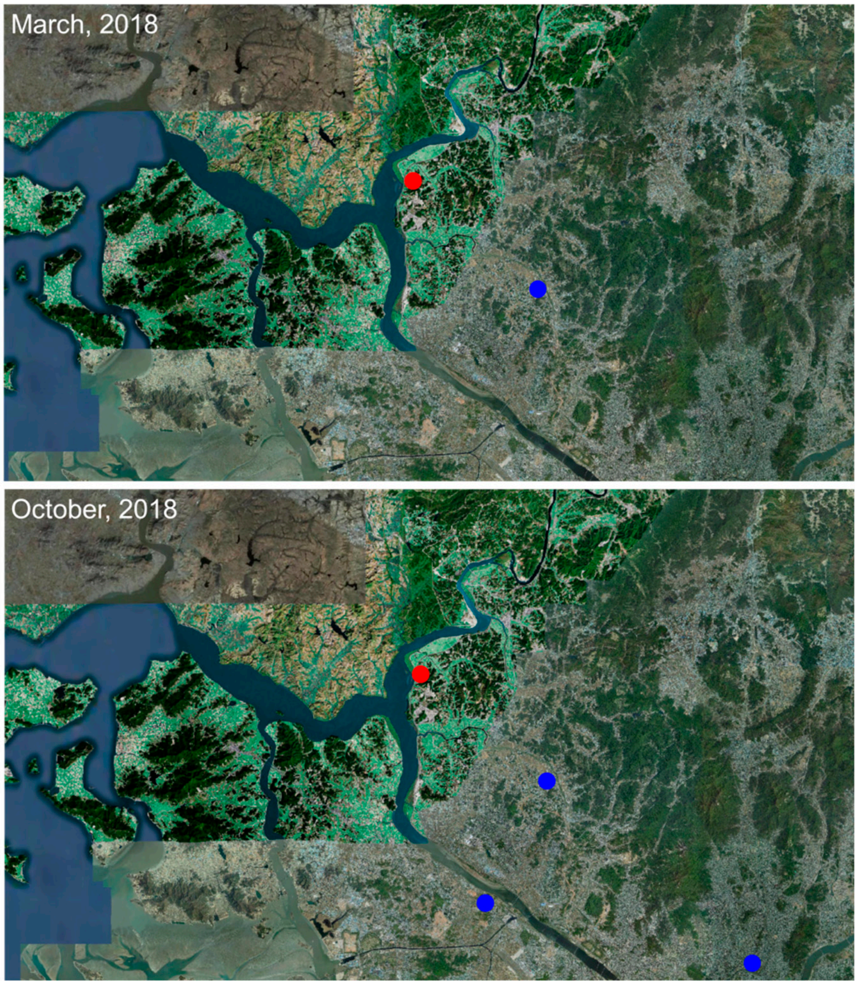

Fig. S3. Location map of Mandarin ducks marked with satellite transmitters in the East Asian Flyways, March and October in 2018. Red dot indicate the isolation place of A/Mandarin duck/South Korea/KNU18-12/2018(H11N9) and blue dots indicate the position of mandarin duck. Map was retrieved from National Institute of Biological Resources, Korea.

Table S2. Detailed NGS analysis

Sample			Sequenced Sample								R_ORF			S_ORF					
Sample Segment#		gene name	# of Pre-processed reads	# of Influenza Virus extracted reads	# of non-Influenza Virus reads	Virus reads %	#M_Reads	Unique Matches	S_Con._bp	%Cov.(S/R)	Length	S_position	E_position	Length	S_position	E_position	method	R_ORF - S_ORF length	Contig Count
K102	1	PB2	23263654	2186688	21076966	0.103748	17148	0.029	2292	1	2280	1	2280	2274	19	2292	standard :segment 8	6	1
	2	PB1, PB1-F2					17999	0.03	2280	1	2274	1	2274	2274	3	2276		0	1
	3	PA, PA-X					16372	0.027	2151	1	2151	1	2151	2151	1	2151		0	1
	4	HA					53303	0.089	1688	1	1689	1	1689	1689	1	1689		0	1
	5	NP					97019	0.162	1505	0.99	1497	1	1497	1506	1	1506		-9	1
	6	NA					4713	0.008	1419	0.9795	1413	1	1413	1395	5	1399		18	1
	7	M2,					333564	0.556	980	0.999	759	1	759	759	1	759		0	1

		M1																	
	8	NEP, NS1					35329	0.059	822	0.9728	693	1	693	690	2	691		3	1

Raw sequence reads were quality trimmed using "trim_galore"(q=20) and non-influenza virus read was removed using Deconseq (iden=60) and python script was used as tool to adjust the amount of data to up to 600,000 read.
Meanwhile a database of only segment 4 (HA), 6 (NA), and 8 (NS1) from the Influenza virus that NCBI was created to provide and align to those of the reference using Gsmapper. (iden=70, ml=40).
ORF was observed with the consensus obtained and adopted a result with an ORF similar to the reference.
As ORF length was different from the reference, sequence error was corrected using ProovRead as previously described [1].
In the NGS reading result, influenza virus reading showed 33.04% reading (influenza: no-influenza = 6404260:19381530) rate and all ORFs were completed.

Table S3. Homology analysis of each gene of A/Mandarin duck/South Korea/KNU18-12/2018(H11N9)

Gene	A/Mandarin duck/South Korea/KNU18-12/2018(H11N9) (GenBank accession # [a])	Virus with the highest homology (GenBank ccession No. [b])	Nucleotide identity [c] (%)	% Nucleotide identity [d](%)
**PB2**	MN596421	A/mallard/Khabarovsk/241/2017 (H10N6)(EPI1333694)	98.9	94.53
**PB1**	MN596422	A/wild bird/Eastern China/1754/2017(H5N3)(MN171456.1)	99.08	92.31
**PA**	MN596423	A/duck/Jiangsu/SE0261/2018(H5N3) (MN171449.1)	99.21	96.72
**HA**	MN596424	A/duck/Kagoshima/KU57/2014 (H11N9)(EPI1057756)<br>A/crane/Kagoshima/KU-T40/2015 (H11N9)(EPI1057748)	97.70	94.99
**NP**	MN596425	A/duck/Aichi/231003/2016 (H8N4)(EPI866835)	99.35	97.26
**NA**	MN596426	A/duck/Kagoshima/KU57/2014 (H11N9)(EPI1057758)	98.00	94.69
**M**	MN596427	A/wild waterfowl/Korea/F7-18/2018 (H4N8)(EPI1566658)	99.80	98.40
**NS**	MN596428	A/avian/Japan/8KI0162/2008 (H3N8)(EPI299270)	99.54	74.89

[a] GeneBank Accession number of eight gene segments of A/Mandarin duck/South Korea/KNU18-12/2018(H11N9)
[b] GeneBank Accession number listed in NCBI and GISAID database
[c] Nucleotide identity of virus showing the highest homology
[d] Nucleotide identity of A/waterfowl/Korea/S353/2016 (H11N9)

a. H1N1 (12 hpi)

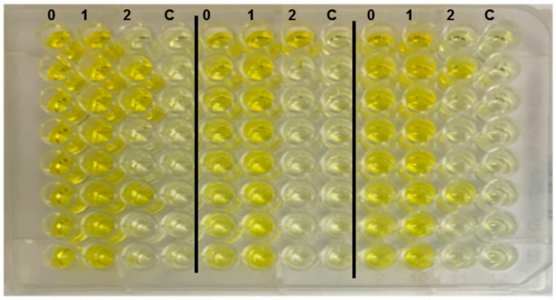

b. H1N1 (24 hpi)

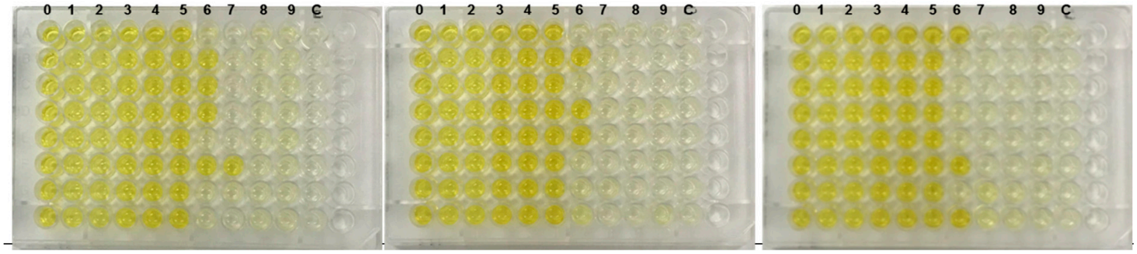

c. H1N1 (36 hpi)

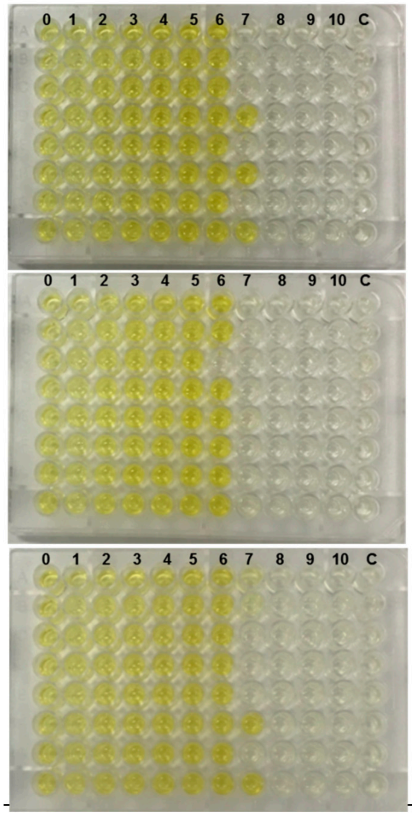

d.H1N1 (48 hpi)

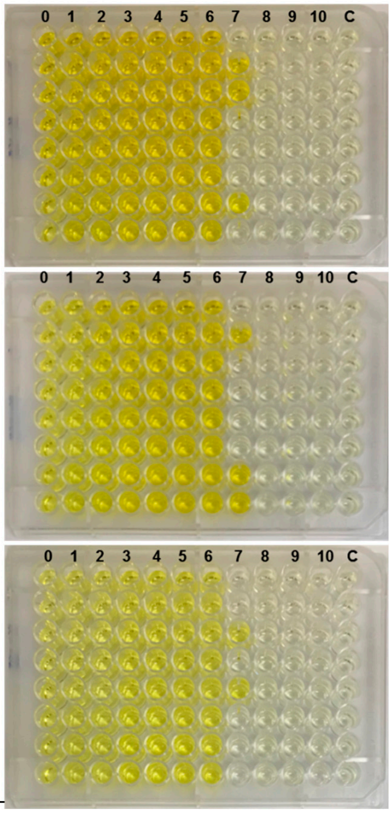

e. H1N1 (60 hpi)

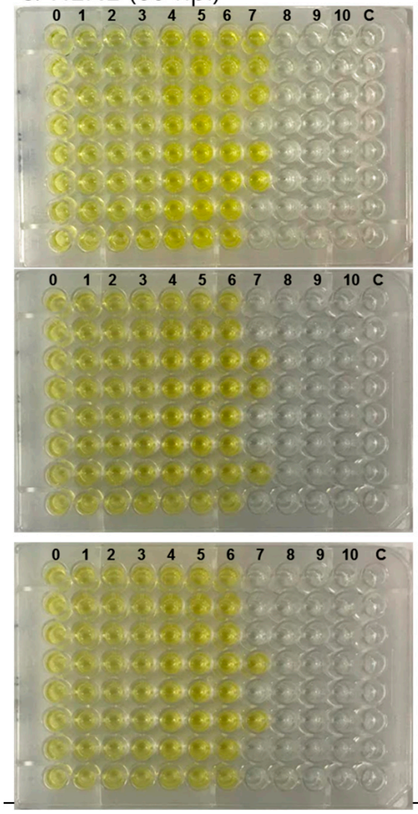

f. H1N1 (72 hpi)

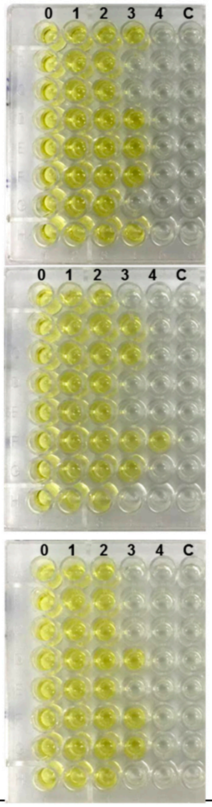

g. H11N9 (12 hpi)

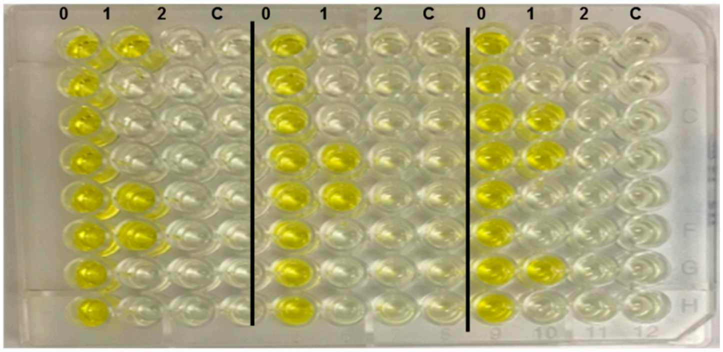

h. H11N9 (24 hpi)

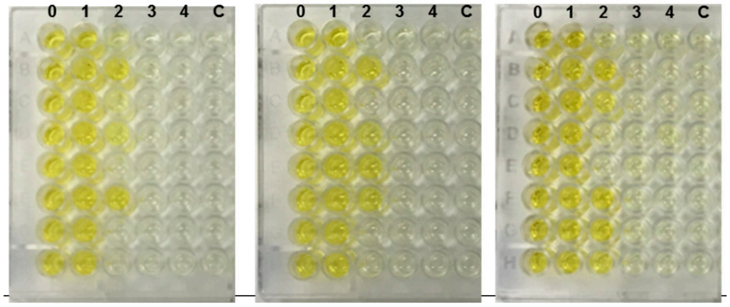

i. H11N9(36 hpi)

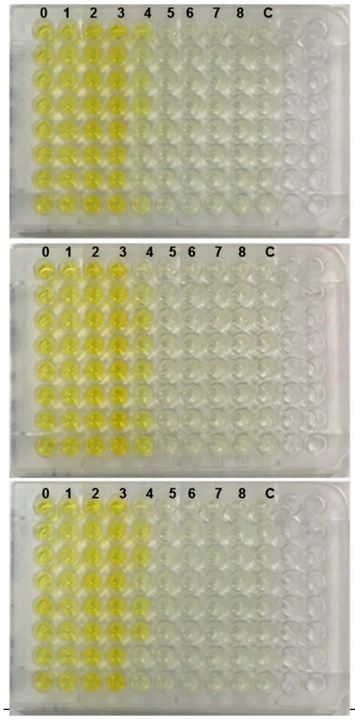

j. H11N9 (48 hpi)

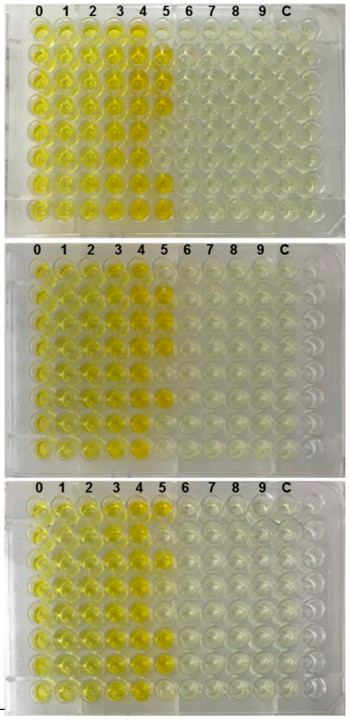

k. H11N9 (60 hpi)

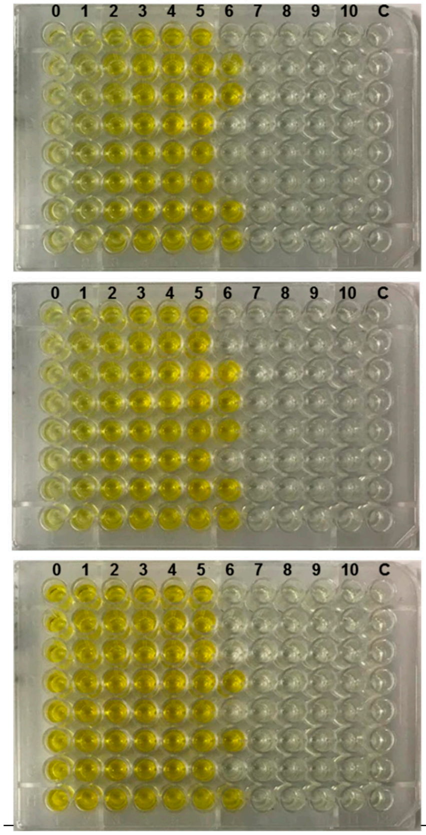

l. H11N9 (72hpi)

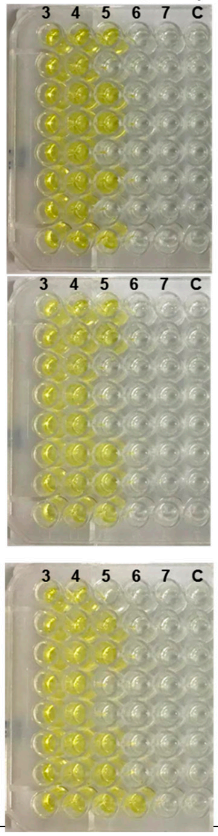

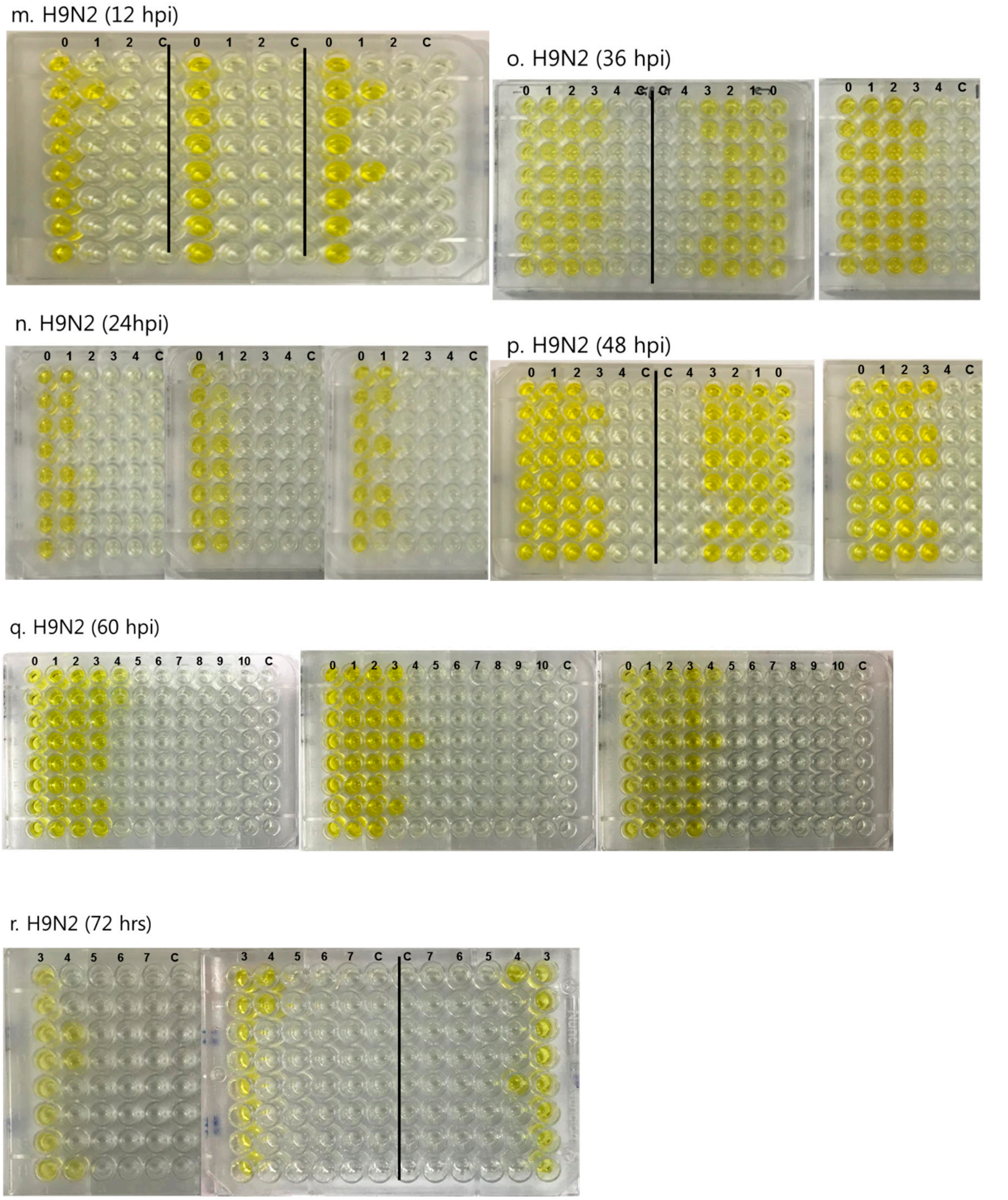

Fig. S4. Raw ELISA data to conduct TCID$_{50}$ assay. Serial 10-fold dilutions of three different viruses H1N1 (a-f), H11N9 (g-l), and H9N2 (m-r) (MOI of initial virus stock: 0.001) infected MDCK for 3 dpi. At every 12 hours post-infection (hpi), cell monolayers were fixed with 10% acetone and blocked with 5% non-fat milk. Cells were washed with PBS and reacted with 0.1 μg/well of anti-influenza nucleoprotein (Medix Biochemica, Finland). After 1 h, secondary Ab in the form of horseradish peroxidase (HRP)-conjugated rabbit anti-mouse IgG (Abcam, Cambridge, UK) was added to each well according to the manufacture's protocol. Stringent washing with PBS-T was performed five times to remove

nonspecific biding and 100 μL of 3,3′,5,5′-tetra methyl benzidine (Sigma-Aldrich) substrate solution was added. 0,10^0- fold dilution of stock; 1,10^1- fold dilution of stock; 2,10^2- fold dilution of stock; 3,10^3- fold dilution of stock; 4,10^4- fold dilution of stock; 5,10^5- fold dilution of stock; 6,10^6- fold dilution of stock; 7,10^7- fold dilution of stock; 8,10^8- fold dilution of stock; 9,10^9- fold dilution of stock; 10,10^{10}- fold dilution of stock; C, mock-infection;

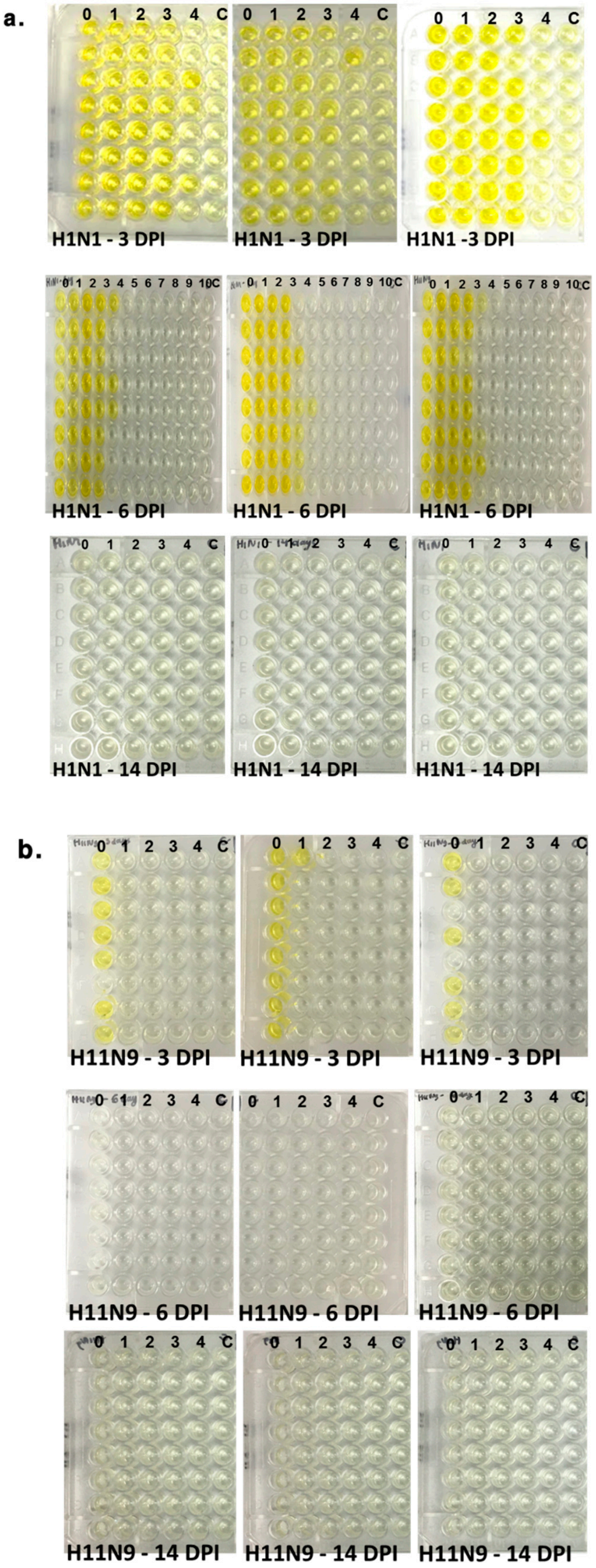
a.
0 1 2 3 4 C
H1N1 - 3 DPI
H1N1 - 3 DPI
H1N1 -3 DPI
0 1 2 3 4 5 6 7 8 9 10 C
H1N1 - 6 DPI
H1N1 - 6 DPI
H1N1 - 6 DPI
H1N1 - 14 DPI
H1N1 - 14 DPI
H1N1 - 14 DPI
b.
H11N9 - 3 DPI
H11N9 - 3 DPI
H11N9 - 3 DPI
H11N9 - 6 DPI
H11N9 - 6 DPI
H11N9 - 6 DPI
H11N9 - 14 DPI
H11N9 - 14 DPI
H11N9 - 14 DPI

Fig. S5. Raw ELISA data to conduct $TCID_{50}$ assay to measure virus titer in lung. H1N1 (a) and H11N9 (b). 0,10^0- fold dilution of stock; 1,10^1- fold dilution of stock; 2,10^2- fold dilution of stock; 3,10^3- fold dilution of stock; 4,10^4- fold dilution of stock; 5,10^5- fold dilution of stock; 6,10^6- fold dilution of stock; 7,10^7- fold dilution of stock; 8,10^8- fold dilution of stock; 9,10^9- fold dilution of stock; 10,10^{10}- fold dilution of stock; C, mock-infection;

Table S4. Genetic similarity with H11N9 strains of different countries

Country	H11N9 Strains	Nucleotide identity							
		PB2	PB1	PA	HA	NP	NA	M	NS
China	A/mallard/Sanjiang/148/2006	92.81%	95.26%	96.05%	95.35%	92.59%	95.61%	96.84%	-*
	A/Oriental White Stork/Zhalong/183/2006	92.68%	95.21%	95.40%	95.17%	91.84%	95.75%	96.74%	-
	A/wild bird/Anhui/S119/2014	97.68%	95.74%	95.03%	95.17%	97.66%	94.83%	97.15%	94.58%
	A/wild bird/Anhui/L306/2014	88.13%	96.09%	90.00%	95.35%	93.99%	94.90%	98.27%	90.80%
	A/wild bird/Wuhan/CDHN01/2015	95.48%	95.22%	95.53%	95.24%	97.66%	94.84%	98.70%	75.11%
	A/wild bird/Wuhan/CDHN22/2015	95.48%	95.22%	95.53%	95.24%	97.60%	94.84%	98.70%	75.11%
	A/wild bird/Wuhan/CDHN173/2015	95.48%	95.22%	95.53%	95.24%	97.54%	94.84%	98.70%	75.11%
	Bean goose/Hubei/SZY200/2016	97.63%	95.25%	96.98%	80.78%	97.26%	94.48%	97.96%	-
Japan	A/duck/Niigata/151015/2016	95.66%	96.20%	94.85%	95.13%	97.91%	94.53%	96.40%	94.32%
	A/duck/Niigata/151019/2016	95.62%	96.20%	94.85%	95.07%	97.91%	94.46%	96.40%	94.32%
	A/duck/Niigata/151014/2016	95.66%	96.20%	94.85%	95.13%	97.91%	94.53%	96.40%	94.32%
	A/duck/Ibaraki/99/2016	88.04%	95.19%	98.68%	95.13%	91.91%	94.88%	95.50%	78.12%
	A/duck/Ibaraki/F99/2016	88.04%	95.24%	98.68%	95.13%	91.84%	94.88%	95.50%	74.91%
	A/crane/Kagoshima/ KU-T40/2015	96.01%	93.80%	96.79%	97.70%	91.45%	97.95%	99.39%	-
	A/duck/Kagoshima/KU57/2014	95.96%	93.85%	96.79%	97.70%	91.38%	98.02%	99.39%	-
	A/duck/Tottori/311217/2014	97.83%	87.41%	95.26%	95.43%	97.39%	94.98%	96.45%	90.60%
Korea	A/waterfowl/Korea/S353/2016	94.53%	92.31%	96.72%	94.99%	97.26%	94.69%	98.40%	74.89%
Brazil	A/ruddy turnstone/ Ilha de Canelas/A008/2008	84.60%	88.16%	89.62%	80.94%	88.78%	87.17%	93.71%	77.65%
	A/ruddy turnstone/Ilha de Canelas/A017/2008	84.73%	88.02%	89.62%	80.88%	88.85%	87.31%	93.61%	77.81%
	A/ruddy turnstone/Ilha de Canelas/A051/2008	84.72%	88.11%	89.62%	81.00%	88.85%	87.24%	93.71%	77.97%

* No similarity

Table S5. Stock titer of virus used in mouse study

Virus	Characteristics		
	EID_{50}/mL	$TCID_{50}$/mL	PFU/mL
KH1N1	$10^{5.17}$	$10^{4.67}$	$10^{4.52}$
H11N9	$10^{7.18}$	$10^{6.2}$	$10^{6.05}$

Table S6. Mouse adaptive mutation site of A/California/04/2009 (H1N1)

Gene	Known mouse adaptive mutation site[3]	A/California/04/2009(H1N1)
PB2	E158G/A	E158
PA	L295P	295P*
NP	D101G	D101
	H289Y	H289
HA	K119N	K119 (136)
	G155E	G155 (172)
	S183P	183P (200)*
	R221K	R221 (238)
	D222G	222G (239)*

*: Mutation site of A/California/04/2009(H1N1)

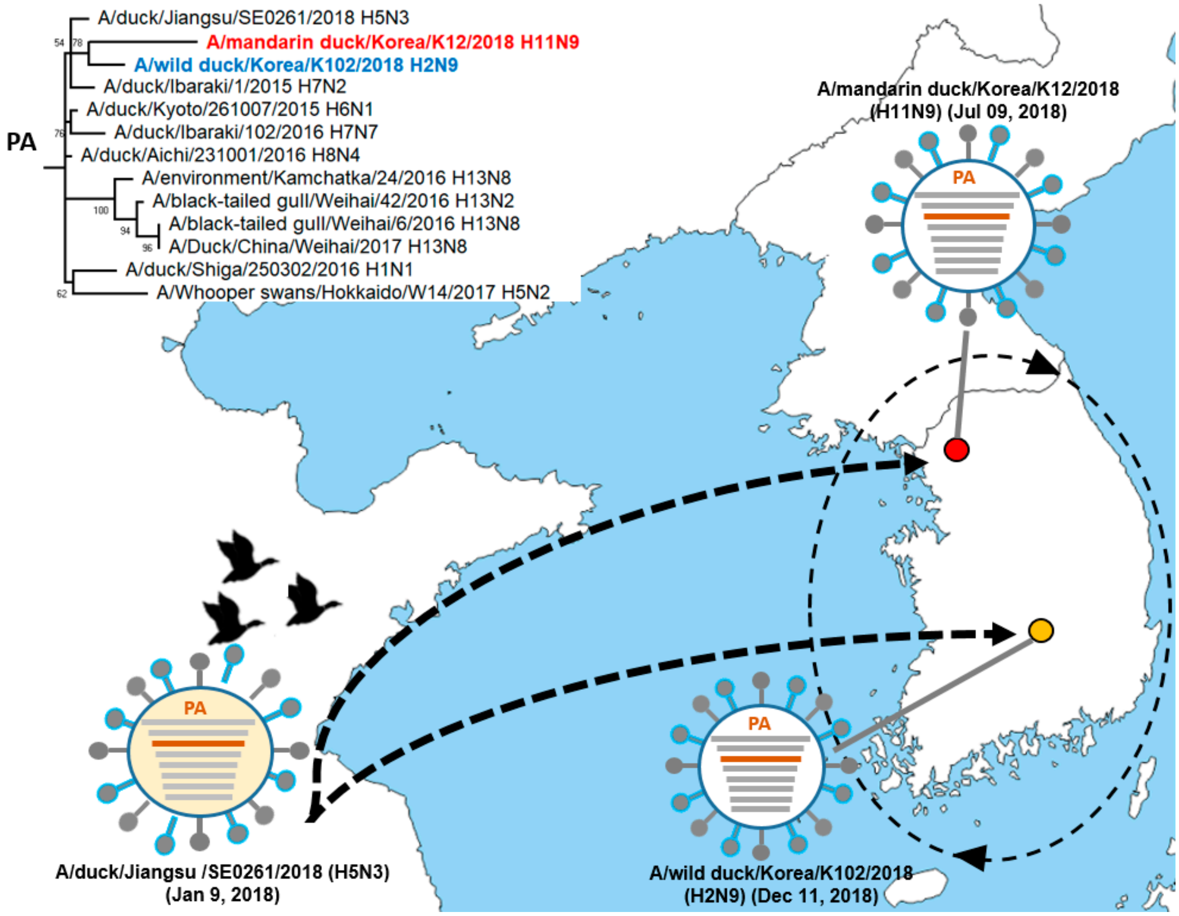

Fig. S6. Close relationship of PA gene of different isolates in Korea in 2018. A/duck/Jiangsu/SE0261/2018 (H5N3) showed the close relation with two different isolates (A/Mandarin duck/South Korea/KNU18-12/2018(H11N9) and A/wild duck/Korea/K102/2018(H2N9) [2].

Reference

1. Hackl, T.; Hedrich, R.; Schultz, J.; Förster, F., proovread: large-scale high-accuracy PacBio correction through iterative short read consensus. *Bioinformatics* **2014,** *30* (21), 3004-3011.
2. Yeo, S. J.; Than, D. D.; Park, H. S.; Sung, H. W.; Park, H., Molecular Characterization of a Novel Avian Influenza A (H2N9) Strain Isolated from Wild Duck in Korea in 2018. *Viruses* **2019,** *11* (11).
3. Ilyushina, N. A.; Khalenkov, A. M.; Seiler, J. P.; Forrest, H. L.; Bovin, N. V.; Marjuki, H.; Barman, S.; Webster, R. G.; Webby, R. J., Adaptation of pandemic H1N1 influenza viruses in mice. *J Virol* **2010,** *84* (17), 8607-16.
